# Scoping review of UK female-firefighters, physical fitness and occupational performance

**DOI:** 10.1093/occmed/kqag042

**Published:** 2026-06-09

**Authors:** Warren Hart, Dan Martin, Daniel Bishop, Daniel Taylor

**Affiliations:** School of Psychology, Sport Science and Wellbeing, University of Lincoln, Brayford Pool, Lincoln, Lincolnshire, LN6 7TS, UK; School of Psychology, Sport Science and Wellbeing, University of Lincoln, Brayford Pool, Lincoln, Lincolnshire, LN6 7TS, UK; School of Psychology, Sport Science and Wellbeing, University of Lincoln, Brayford Pool, Lincoln, Lincolnshire, LN6 7TS, UK; School of Psychology, Sport Science and Wellbeing, University of Lincoln, Brayford Pool, Lincoln, Lincolnshire, LN6 7TS, UK

## Abstract

**Background:**

Firefighting is a physically demanding occupation with sex-neutral minimum physiological performance requirements. Whilst 9% of UK firefighters are female, there has been no systematic review of scientific literature concerning their role-specific physiology and performance.

**Aims:**

To summarise UK-based literature concerning female firefighters within four key areas: physical demands of operational tasks, testing and training, injuries, and influence of female-specific physiology on training task performance and injury.

**Methods:**

This systematic scoping review adopted the 5-step framework for conducting scoping reviews from Arksey and O’Malley [34], alongside The SPIDER tool to identify and summarise peer-reviewed data for female UK firefighters.

**Results:**

Four papers were identified, including 31 females. Hose running imposed the highest physical demand on females (44.0 ml·kg−^1^·min^−1^). Mean maximal aerobic capacity values were 44.4 ± 5.5 ml·kg^−1^·min^−1^ (directly measured) and 46.9 ± 6.7 ml·kg^−1^·min^−1^ (estimated), assuming normal distribution, approximately 35% of the population would not meet the threshold of 42.3 ml·kg^−1^·min^−1^. Mean Firefighter Simulation Performance of females was 7.4 seconds slower than the passing requirements. No published data were available involving fitness interventions, injuries or female-specific considerations.

**Conclusions:**

There is a paucity of UK-based research exploring the assessment, maintenance and development of physical fitness and occupational performance in female firefighters. Further research is needed to expand the current evidence-base relating to the occupational demands of female firefighters and physical fitness testing to inform policies by using a more representative evidence-base. Additionally, this work highlighted the absence of published research related to fitness training, injury surveillance and female-specific considerations.

## Teaser

A 70% increase in representation over the last 10 years means that female firefighters now make up ∼9% of the UK fire and rescue sector. However, there has been no systematic review to date of physiological fitness and performance research relating to female firefighters in the UK. This scoping review provides an assessment of the current physiological performance data and associated demands of occupational tasks in female firefighters within the UK fire and rescue sector.

Key learning points **What is already known about this subject:**

- Firefighting is a physically demanding occupation with minimum physiological performance requirements needed to meet the wide-ranging demands of operational tasks and environments; UK occupational standards are sex neutral; firefighters are required to meet a minimum standard of 42.3 ml·kg^−1^·min^−1^ at least annually.
- Aerobic capacity and muscular strength are highlighted as the main determinants of successful performance, with task-related minimum aerobic values highlighted as the main predictor of successful outcomes during firefighting tasks.
- A 70% increase in representation over the last 10 years means that female firefighters now make up ∼9% of the UK fire and rescue sector; therefore, there is a need to understand female firefighters’ physiological performance.

**What this study adds:**

- Only four studies have published data relating to UK female firefighter physical fitness and occupational performance; no research has been published on the fitness development and training, injury frequency, type, duration or mechanisms, or female-specific physiology of UK-based female firefighters.
- Aerobic capacity has only been reported in 31 female UK firefighters and is lower when directly measured (44.0 ± 4.4 ml·kg^−1^·min^−1^) compared to when estimated (46.9 ± 6.7 ml·kg^−1^·min^−1^) and 2.5-2.6 ml·kg^−1^·min^−1^ lower than male counterparts.
- In a very limited sample of female firefighters, it has been shown that the aerobic demands of simulated firefighting tasks are similar between males and females, however female firefighters performed slower in firefighter simulation assessments.

**What impact does this have on practice or policy:**

- This study highlights that there is very limited research on which to base the practice and policy for UK female firefighters in relation to physical fitness and occupational performance.
- There is a need for further studies with a more representative population to ensure alignment between the physical demands of tasks/duties and testing criteria that are suitable for the whole firefighting population, rather than primarily based upon male data.
- Future research should study injury incidence and female-specific considerations, while ensuring the validity of testing approaches is considered across a representative sample.

## Introduction

Firefighting is a physically demanding occupation with minimum physiological performance requirements needed to meet the wide-ranging demands of operational tasks and environments [1–4]. To ensure all firefighters can meet these occupational demands, national guidance requires fire services across the UK to evaluate fitness during the recruitment process and then annually throughout their career [4,5]. There is an expectation that firefighters maintain sufficient levels of fitness to be operationally effective, using the facilities (e.g. gyms and allocated personal training time during shifts) and associated support mechanisms (e.g. fitness officers, exercise physiologists) provided by their service or at the station. Whilst the occupational standards for testing and training processes remain sex and age-neutral, the demographic profile of operational firefighters in the UK includes a growing proportion of females across a wide range of ages. The number of female firefighters in the UK has increased by 70% in the last ten years (from 1755 to 2987; 9% of current firefighters; [6]), and the service has a long-standing aim to grow these numbers further so that females make up at least 15% of the total UK fire and rescue workforce [7]. Given these points there is a need to establish how the physical fitness and role-specific performance of female firefighters can be supported across the career span through effective evidence-based testing and training practices.

It is already widely accepted across a range of physically demanding occupations (e.g. law enforcement, military) and sport settings (e.g. football, rugby, endurance sports) that differences exist between sexes in relation to physiological performance [8,9], injury rates and mechanisms [10,11], performance of tasks [12,13], age-related physiological changes [14], and adaptations to physical training programmes [15–17]. Such differences are also likely to exist between male and female firefighters, with evidence that operational tasks (e.g. carrying, lifting, equipment runs) require the average female to perform at a higher percentage of maximal capacity than their male counterparts [4]. This may, in turn, be associated with earlier or greater levels of fatigue development which may have implications for firefighter effectiveness [18] Furthermore, these differences may be exacerbated by aging (and extended retirement ages) given that firefighters’ fitness typically reduces across their career [19,20]. As such, there are a number of female-specific considerations which may have an impact on the fitness of operational firefighters, such as the menstrual cycle and associated menstrual disorders (e.g. dysmenorrhea, menorrhagia), pregnancy and post-partum periods, and the menopause (with ∼17% of female UK firefighters reported as perimenopausal or menopausal [21–23]). Given that each of these biological phases has been broadly associated with changes in muscular strength, aerobic capacity performance and development [24,25], a review of available evidence is certainly warranted in relation to female firefighters and the evaluation, maintenance and development of their physical fitness over the course of their career. Such a review would improve understanding about how the testing, training, and support of adequate fitness levels for female firefighters within the UK can be optimised, rather than the potentially flawed ‘one-size-fits-all’ approach currently adopted across all ages and genders within the service.

A number of studies have explored factors related to the physiological fitness and performance of female firefighters in non-UK services, including studies on task demands and time performance [26,27], specific physiological variables related to performance [28,29], impact of heat [30], impacts of female-specific training interventions [31] and the variations in female and male injury type and rates [32]. This body of work provides broad support for overarching sex differences in firefighters and illustrates that the demand for female-specific evidence exists across the global firefighting sector. However, there are established differences in environment, equipment, demands, policies, culture, working hours and shift patterns, fitness standards and methods of testing, support and provisions, as well as academic research between UK fire service and international counterparts. Consequently, a UK-specific scoping review is essential to ensure that future provisions are evidence-based, operationally relevant, and tailored to the unique demands of the UK Fire and Rescue Service [3,21].

Based on the points outlined, there is a need to better understand how the physical fitness and role-specific performance of female firefighters in the UK can be supported across their careers through effective evidence-based testing and training practices, particularly during key biological phases they are likely to experience. Until now there has been no formal review of the evidence relating to physical fitness and occupational performance in UK female firefighters. Therefore, the aim of this study was to summarise the current research evidence relating to the physical fitness and role-specific performance of UK female firefighters. More specifically, this study provides a systematic review of research to date relating to four key elements: (i) the physical demands of operational tasks or duties in female firefighters; (ii) physical fitness testing and training intervention data in female firefighters; (iii) the frequency, type, duration and mechanism(s) of injuries reported by female firefighters; (iv) the effects of physiological changes specific to female firefighters (i.e. menstrual cycle, menopause, post-partum) on fitness, operational performance, physical training or injury occurrence.

## Methods

The scoping review was pre-registered with Open Science framework. The research team developed a review framework to help screen and validate relevant current literature [33]. This systemic scoping review adopted the SPIDER (Sample, Phenomenon of interest, Design, Evaluation and Research Type) 5-step process for scoping reviews [34] (Table 1), employing recommendations for enhancing the reviewing process [35].

**Table 1:** The study search review questions using the SPIDER Tool [36]

Sample	Operational (i.e. active) female firefighters of any age, rank and must be UK fire and rescue service.
Phenomenon of interest	The physical demands of operational tasks or duties. Physical fitness testing and training Frequency, type, duration, and mechanism(s) of injuries Female-specific physiological changes (i.e. menstrual cycle, menopause, post-partum) on firefighter fitness, operational performance, physical training, or injury occurrence.
Design	Published literature of any research design must contain quantitative data related to physiological/physical performance, fitness testing information, physical training of female firefighters, injury or female-specific physiological changes, and firefighting task specific performance.
Evaluation	Distribution of discipline-specific research, study characteristics, sample characteristics must have female date presented separately, key findings must show female data separately.
Research type	Quantitative peer-reviewed studies, educational theses, and conference proceedings.

The search aimed to identify literature related to firefighting performance, fitness, and injuries in UK-based female firefighters. The first search was conducted on the 1^st^ of September 2024. The databases chosen for the search were Medline, PubMed, SPORTDiscus, Web of Science, CINAHL and Embase. Hand searching of articles also took place and was conducted using Google Scholar, from the reference list of articles and a search of UK based fire and rescue services websites. More specifically, the search terms used were as follows:

S1 Full text “woman*” OR “women*” OR “female*”; S2 Full text “fitness” OR “physical” OR “health” OR “physiological” OR “aerobic” OR “strength” OR “performance” OR “exercise” OR “training” OR “Physical performance” OR “Functional Tasks” OR “Firefighting Tasks” OR “Interventions” OR “Exercise Interventions” OR “Heat” OR “Occupational Tasks” OR “Menopause” OR “Pre Natal” Or “Post Natal” OR “Return to work” OR “Heat” OR “Self-Reported” OR “Time off Work” OR “Minimum physical performance” OR “Minimum Demands” OR “Selection Standards”; S3 Full Text (“firefight*” OR “firefighter*”); S4, S1 AND S2 AND S3.

The scoping review included all peer-reviewed published academic literature, conference proceedings, alongside post-graduate dissertations and theses. The articles were required to include quantitative data related to the phenomena identified in Table 1. Additionally, the sources must have reported on a female-only sample or reported female data separately from the male data. All female data must be from operational firefighters within the UK fire and rescue sector. Only studies that were written or translated into English were included in the scoping review, to ensure there was no misinterpretation of information following translation.

All screening was conducted using Covidence (Covidence, Melbourne, Australia., 2024) screening software [37]. The initial title screening of articles was conducted by 1^st^ author (WH) and 2^nd^ Reviewer (DM), and a 3^rd^ reviewer (DB) had the final decision on any disagreements. All three reviewers then conducted abstract reviews due to the low number of articles returned, with an inclusion-exclusion decision made by consensus.

Data extraction was conducted by the 1^st^ author (WH) from reading the full text. The data extracted was in line with the research questions: author, title, publication date, study design, country of research origin, sample size, sex specific data and key findings, including whether these were sex-specific or independent of sex. Upon completion, the reviewing team then verified the information extracted by reading the full text articles. Weighted means (${\bar{x}}_{w}=\sum (w_{i}x_{i})/\sum w_{1}$) and standard deviations ($\sigma_{w}\sqrt{\sum w_{i}{\left(x_{i}-{\bar{x}}_{w}\right)}^{2}∕\sum w_{i}}$) were calculated to normalise data according to sample size, where appropriate.

## Results

**Figure 1:**
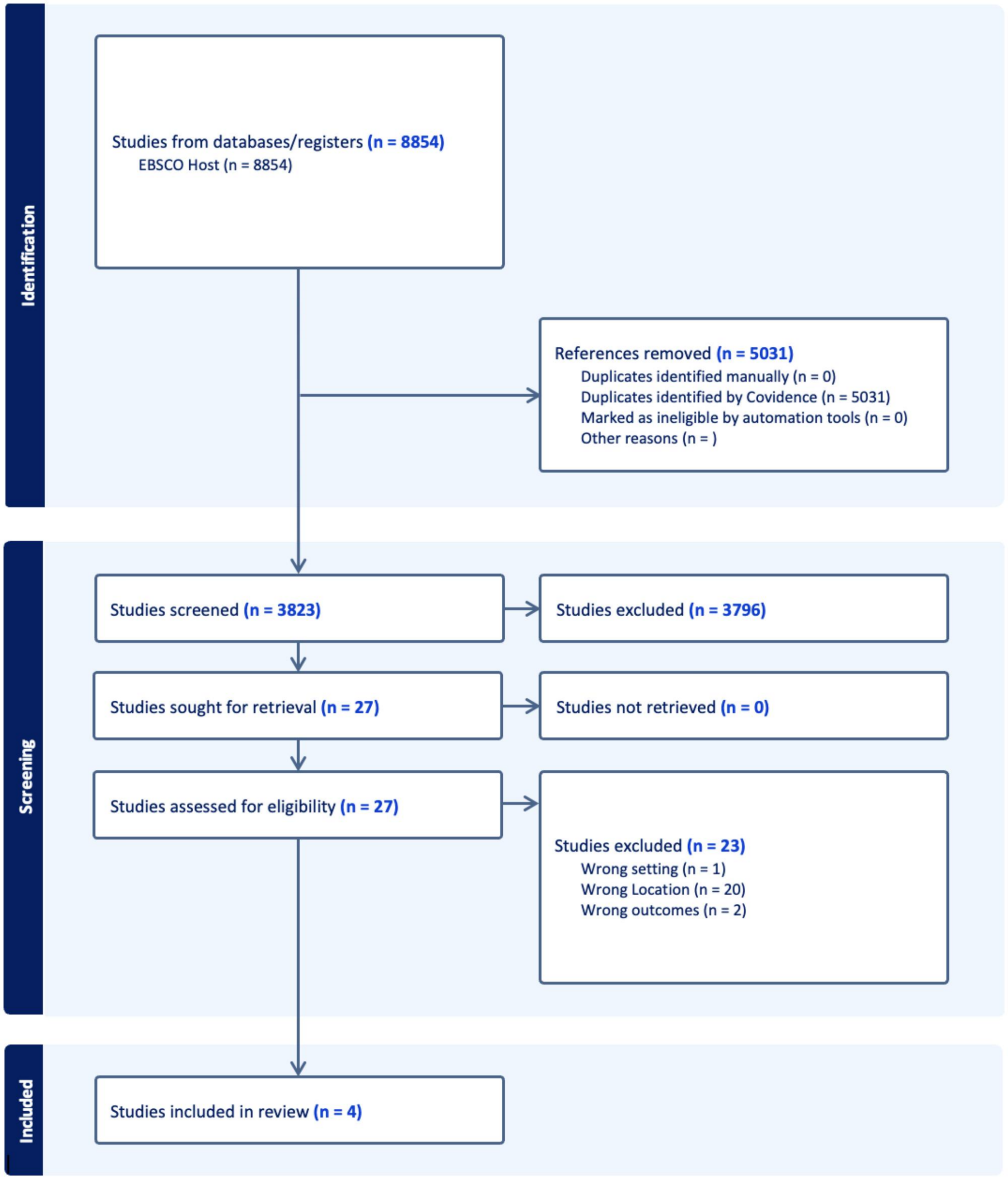
PRISMA flowchart of the scoping review process used [38].

After removing duplicates, a total of 3823 articles were identified from electronic databases searches and hand screening. After conducting an abstract and title screening phase, 27 articles were included in the full text screen, 23 articles removed at the full text screening stage were due to wrong location (*n* = 20), wrong setting (*n* = 1), wrong outcomes (*n* = 2; Figure 1).

The sample consisted of four studies summarised in Table 2. These studies included a total of *n* = 181 participants, of which *n* = 31 (17%) were females and *n* = 150 (83%) were males, all of whom were operational firefighters. There were no studies with female-only data sets.

**Table 2:** Overview of the studies included in the scoping review

Study	Aims	Study design	Number	Males	Females	Findings
Siddall *et al.*, [3]	Develop role-related aerobic capacity standards for UK firefighters, established from monitoring performance on previously agreed tasks required in operational scenarios.	Reliability and validity	62	50	12	An aerobic capacity of 42.3 ml·kg^-1^·min^-1^ was found to be the minimum standard required when all tasks were taken into consideration and intensity to time values were analysed.
Stevenson *et al.*, [4]	Design a functional firefighter fitness assessment, then explore the validity and reliability of the firefighter simulation assessment.	Reliability and validity	69	64	5	FFST completion time had a strong inverse relationship with VO_2_max (*r* = 0.73) and fat mass. FSST had a reasonable validity and good reliability (*r* = 0.84); however, a high prediction error indicates that this should not be used as an assessment of aerobic fitness for firefighting.
Hart *et al.*, [5]	Investigate the validity and reliability of predictive measures for aerobic capacity and strength in comparison to directly measured methods. Additionally, understand the influence of performance variables on FFST performance.	Reliability and validity, cross-sectional	22	19	3	Predictive measures of lower limb strength (Flex) are a valid assessment of lower body strength. CST is not a valid assessment of aerobic capacity as LOA are large (12.2 ml·kg^-1^·min^-1^). The FFST is not a valid assessment for establishing aerobic capacity as other factors such as lower limb strength, age and body mass contribute strongly to performance.
Stevenson *et al.*, [39]	Establish the impact of cardiorespiratory fitness levels and the associated performance in high-rise task performance.	Cross sectional	28	17	11	Those with higher levels (>42.3 ml·kg^-1^·min^-1^) of cardiorespiratory fitness were 219 seconds quicker in high-rise associated task performance compared to those with lower fitness. Lower heart and breathing frequency were also associated with higher levels of fitness, thus reducing risk in firefighting tasks.
4	–	–	181	150	31	–

CST, chester step test; FFST, firefighter simulation assessment;Flex, laser optic velocity tracker; LOA, limits of agreement; VO_2_max, maximal oxygen uptake.

Four UK-based studies had anthropometric data from female UK female firefighters (*n* = 31; Supplementary Table 3). Female pooled data showed mean values of age 34.4 ± 7.3 years, height 170.2 ± 4.5 cm, body mass 67.0 ± 8.1 kg and a BMI of 23.2 ± 2.6 kg·m^−2^. Females were 5.2 years younger, 8.5 cm shorter, 19.7 kg lighter with a BMI of 3.7 kg·m^−2^ less than male firefighters.

Two studies (3; *n* = 12, 5; *n* = 3) reported the physical demands of simulated firefighting tasks for females (Supplementary Table 4). Siddall *et al.* [3], assessed the highest oxygen consumption of 9–12 female participants across 5 simulated firefighting tasks, directly measured using metabolic carts. Compared to males, females had a 4.0 ml·kg^−1^·min^−1^lower oxygen demand for hose run and a 3.0 ml·kg^−1^·min^−1^ greater oxygen demand for equipment carry, although there were no significant differences between sexes. Hart *et al.* [5] found that despite females (*n* = 3) recording 8% slower (46 seconds) performance completion times during simulation tasks when compared to male counterparts (n = 19), both sexes were working at 96% of heart rate max.

Aerobic capacity data was measured in four studies (Table 3). Two studies estimated aerobic capacity via either a sub-maximal treadmill ramp assessment (3, *n* = 12) or the Chester step test (5, *n* = 3). Three studies directly measured aerobic capacity using expired-gas analysis during treadmill-based maximal ramp tests (4; *n* = 5, 5; *n* = 3, 39; *n* = 11).

**Table 3:** Sex specific directly measured (indirect calorimetry) and estimated aerobic capacity values (calculated using heart rate or performance equivalent) and heart rate values [3, 4, 5, 39]

	Directly measured absolute VO_2_max (L^.^min^-1^)		Directly measured relative VO_2_max (ml·kg^-1^·min^-1^)		Estimated relative VO_2_max (ml·kg^-1^·min^-1^)		HRM (beats·min^-1^)	
	Male	Female	Male	Female	Male	Female	Male	Female
Siddal *et al.*, [3]	–	–	–	–	50.9 ± 6.3	46.6 ± 7.4	–	–
Stevenson *et al.*, [4]	–	–	48.0 ± 9.2	45.5 ± 4.2	–	–	–	–
Hart *et al.*, [5]	4.3 ± 0.6	3.3 ± 0.3	48.0 ± 5.6	50.1 ± 2.7	46.7 ± 8.3	47.9 ± 3.4	189.0 ± 8.0	194.0 ± 6.0
Stevenson *et al.*, [39]	–	–	45.2 ± 6.0	41.8 ± 4.3	–	–	–	–
**Pooled mean**	4.3	3.3	47.0	44.4	49.7	46.9	189.0	194.0
**SD**	0.6	0.3	8.2	5.5	7.1	6.7	8.0	6.0

HRM, heart rate max; VO_2_max, maximal oxygen uptake.

Pooled means estimated aerobic capacity (*n* = 15) were 46.9 ± 6.7 ml·kg^−1^·min^−1^, which was 2.9 ml·kg^−1^·min^−1^ lower than male counterparts [3,5]. Pooled means of directly measured aerobic capacity (*n* = 19) were 44.0 ± 5.5 ml·kg^−1^·min^−1^, which was 2.6 ml·kg^−1^·min^−1^ lower than male counterparts. As such, directly measured aerobic capacity was 2.5 ml·kg^−1^·min^−1^ lower than estimated values in female firefighters.

Two studies (4,5; female *n* = 9, male *n* = 83) included time to completion data for the firefighter simulation test (FFST). Female pooled mean completion time was 678.4 ± 62.9 seconds (Supplementary Table 1) which is 7.4 seconds (1%) slower than the safety critical pass criteria (4; 671 seconds). Assuming normal distribution, this reflects a pass rate of 44% in the FSST. Performance times for females were 81.8 seconds (13%) slower than male counterparts (pooled mean = 595.7 ± 70.6 seconds).

No sex-specific statistical comparisons were made in these studies. However, Hart *et al.* [5] found that aerobic capacity and lower limb strength were stronger predictors of successful FSST performance in a model including female participants compared to a model using male-only data.

One study [5] explored firefighter lower limb strength via direct and estimated (Flex) one repetition max (1RM) strength in a squat movement (Supplementary Table 2). Data from one female participant was reported in the study (1RM directly measured = 92.5 kg and 1.3 kg·kg^−1^; estimated = 90.6 kg and 1.4 kg·kg^−1^). These values were 48.7 kg (42%) and 0.2 kg·kg^−1^ (14%) lower than male counterparts (*n* = 9).

No papers included any data related to the fitness development and training specific to female firefighters.

No papers included any data related to the frequency, type, duration or mechanism of injuries in UK female firefighters.

No papers included any data related to female-specific physiological changes (i.e. menstrual cycle, menopause, post-partum) on firefighter fitness, operational performance, physical training, or injury occurrence in UK female firefighters.

## Discussion

The aim of this scoping review was to summarise the current research evidence relating to the physical fitness and role-specific performance of UK female firefighters, specifically with regards to (i) the physical demands of operational tasks or duties; (ii) physical fitness testing and training intervention data; (iii) injury frequency, type, duration and mechanism(s); and (iv) the effects of physiological changes specific to females (i.e. menstrual cycle, menopause, post-partum) on fitness, operational performance, physical training or injury occurrence. To the knowledge of the authors this is the first systematic review of evidence relating to these topics. The main finding from this review is that very limited research evidence to date has been published relating to these aspects of female firefighter physical fitness and physiology with no published research to date exploring training interventions, injury epidemiology or female-specific considerations. More specifically, only four studies in total were eligible to be included in the current review. The 4 studies only account for a small representation (*n* = 31, 1%) of the total 2987 current operational UK female firefighters employed within the UK fire and rescue sector [6], with the sample of female firefighters included in the studies relatively young (34.4 ± 7.3 years) and lean (BMI = 23.2 ± 2.6 kg·m^−2^), and therefore data are indicative and may not be entirely representative of the female firefighter workforce.

The two studies to examine the physical demands of occupational tasks in female firefighters measured a variety of simulated firefighting tasks across a total of 15 female participants [3,5]. Based on oxygen uptake measurement, Siddal *et al.* [3] established that hose running (44.0 ml·kg^−1^·min^−1^) imposed the highest physical demand on females relative to other tasks (e.g. equipment carry, stair climb, casualty drag) and wildfire suppression (29 ml·kg^−1^·min^−1^) was the least demanding. In relation to FFST performance, Hart *et al.* [5] observed mean heart rates above 95% of estimated maximum values in female participants. These trends are in keeping with data reported for male firefighters in terms of relative demand across firefighting tasks, although it is evident that a wider range of physiological measures (e.g. lower limb strength, directly measured aerobic capacity, muscular fatigue), across a higher number of female participants (and more firefighter-specific tasks) is needed to evidence the physical demands of firefighting tasks in females.

The physical performance standard of firefighting varies internationally, given the occupational tasks that each service is required to perform; however, these tasks all require physical performance to complete tasks successfully. The physiological variables of aerobic capacity and muscular strength are highlighted as main determinants of successful performance with task-related minimum aerobic values highlighted as the main predictor of successful outcomes during firefighting tasks [1–3,29,40].

As previous literature has highlighted, it is essential to understand the physiological demands of firefighting tasks to ensure the safety of individuals, co-workers, and the public, which led to the development of minimum UK performance standards [3]. However, as highlighted in this review, there is a lack of female-specific data, with the evidence underpinning the physiological testing based on only 12 female participants. For a safe/effective testing criterion, this needs to relate to the known physical demands of key operational tasks/duties for the population, given the sex-differences in body size, strength and aerobic capacity [8]. As such, there is a need to have further studies of a representative population to ensure alignment between the physical demands of tasks/duties and testing criteria that are suitable for the whole firefighting population, rather than primarily based upon male data.

There were only eight female participants across two studies assessing performance in the FSST. The weighted mean female completion time was 7.4 seconds above the maximum time allowed for successful performance (671 seconds) [4,5], with mean times of participants (*n *= 5) from Stevenson *et al.* [4], being 35.0 seconds slower than the required performance time. Sex differences in pooled mean data showed females were 81.8 seconds slower in completion of the tasks in comparison to male counterparts. Hart *et al.* [5] established that lower limb strength was an influencing performance variable in quicker performance times alongside aerobic capacity, BMI, and age. However, there was limited female data to draw conclusions specific to sex, so findings established from mixed groups only were analysed. Future research would benefit from understanding which factors influence FSST performance in females to aid the development of targeted support for female firefighters to meet the passing criteria.

Previous research has highlighted that aerobic capacity is the main predictor of firefighter performance and, as such, firefighters are required to meet a minimum standard (42.3 ml·kg^−1^·min^−1^ at least annually [3–5]. Female pooled mean aerobic capacity values (ml·kg^−1^·min^−1^) were 44.4 ± 5.5 (directly measured) and 46.9 ± 6.7 (estimated). Therefore, assuming normal distribution, approximately 35% (when directly measured) and 27% (when estimated) of females in the population would be expected to score under the minimum pass threshold of 42.3 ml·kg^−1^·min^−1^. While estimations of pass rates based upon an assumed normal distribution should be interpreted with caution in small sample sizes, these findings highlight two main points. Firstly, there is a need to improve the provision to support female firefighters to be able to meet the minimum standards. Watkins *et al.* [21], previously reported that 94% of 253 UK female firefighters have concerns around meeting the minimum physical standards for both aerobic capacity and strength, evidencing that this is an area that requires significant development within the service. Secondly, findings from these studies highlight concerns around the application of estimated assessments amongst female firefighters. Estimated aerobic capacity values were 2.5.1 ml·kg^−1^·min^−1^ greater compared to directly measured values across the whole sample, and 2.2 ml·kg^−1^·min^−1^ greater when the same participants were compared in two both assessments [5]. Consequently, inflated estimates may overstate aerobic capacity, allowing firefighters to pass the criterion test without meeting the actual physiological demands of operational duties These potentially inflated estimated measures should be considered when conducting future research with directly measured methods considered to offer greater validity in data and research findings, however, increased data, especially using a more diverse sample, would increase empirical evidence [5].

In the included studies, female participants had a pooled relative aerobic capacity that was 2.6 ml·kg^−1^·min^−1^ (−6%; directly measured) and 2.8 ml·kg^−1^·min^−1^ (6%; estimated) lower than male counterparts. This was despite Hart *et al.* [5], reporting a greater directly measured (+ 2.1 ml·kg^−1^·min^−1^) and estimated (Chester Step Test) (+1.2 ml·kg^−1^·min^−1^) relative aerobic capacity in female participants. However, this is likely explained by the respective body composition (BW/BMI) of females (66.3 kg, 22.7 kg·m^−2^) and males (90.3 kg, 27.1 kg·m^−2)^ as directly measured absolute aerobic capacity was 1 L·min^−1^ (+26%) greater in males compared to females. Therefore, future research should look at publishing data in absolute volumes (L·min^−1^) as well as relative volumes (ml·kg^−1^·min^−1^) to allow a greater understanding of aerobic function, particularly when comparing sexes [41]. British Armed Forces research has explored female performance in physical employment standards and identified the modifiable and non-modifiable physiological characteristics. Findings from female performance (*n* = 46) highlighted that improving modifiable specific physiological variables such as (VO_2_peak), lower limb strength, and upper body power had a positive increase in task performance, such as 2 km run, repeated lift and carry, and water can carry [15]. Given these findings, future firefighting research should look at establishing a better understanding of female-specific task performance and interventions to develop modifiable variables such as aerobic capacity, upper and lower body muscular strength.

No studies were found that explored the effect of training interventions on female firefighter fitness. Firefighters are a unique population who are encouraged to train during their workday and are required to undergo regular fitness testing, with a high proportion of services having access to fitness equipment and professionals. Customised interventions related to the demands of firefighting and the balance of shift work could be designed; however, no research seems to be available to assess the effectiveness of interventions or other factors such as compliance, adherence, and accessibility of resources. In the general population, it has been shown that High Intensity Interval Training (HIIT) has similar outcomes in females and males [42], while endurance training is less effective in females compared to males [43], which might have implications for effective exercise prescription, especially if there is a need to minimise time spent exercising whilst on shift. Concerning strength, research shows that females gain less absolute strength compared to males, but similar relative strength [44]. However, given the non-sex-specific performance requirements, this might mean an increase in volume of training is required for females, given the typically lower baseline of strength associated with physiological impacts previously discussed [8,45].

The review identified no papers on injuries of female firefighters within the UK. In-keeping with international research further work is needed to establish and review the occurrence and characteristics of exercise and training injuries [46,32], type and mechanisms [47,48], and understand the experience of injuries [49] Research could also draw from other tactical occupations, such as optimised physical training [50,51] reduce musculoskeletal injuries (MSKIs) [52], assess injury risk [52], and understand sex specific injury types [51].

The British Armed Forces have explored reducing the risk of injury in females by understanding the mechanisms contributing to injury. A survey of 3022 participants found that that 20% of the sample had suffered a bone stress injury and that 2% had suffered a bone stress injury in the last 12 months; 40% had a time-loss musculoskeletal injury in the last 12 months, and 11% were medically downgraded due to a musculoskeletal injury. Associations highlighted that eating disorders and low energy availability were the main contributors to musculoskeletal injuries, thus highlighting risk in the female military population [53]. The Armed Forces have also explored the sex difference of injury rates during training (direct entry and officer training); findings showed a similar overall sex injury risk rate but highlighted that females are at 52% and 72% respectively, increased risk of stress fractures, thus highlighting the need for sex specific screening and interventions [54]. Future firefighting population research should identify female-specific risk factors in firefighting tasks and reducing the risk of injury; and understanding the demands of the role and establishing sufficient, progressive and safe training to enable female to meet these demands [55].

The review identified that there was no published research exploring the effects of female specific physiology (e.g. menstrual cycle, hormonal contraceptives, pregnancy, menopause) on the fitness, performance, training or injury occurrence of UK-based female firefighters. Given the increase in female firefighters and the desire to increase the diversity of the workforce there is a growing demand on understanding female physiology across a life cycle in more depth. International research within the firefighting population has highlighted concerns around achieving physical performance standards and the risk of injury with increased age [28,21]. Other tactical occupations have explored female-specific physiology and anatomy in relation to sex-specific training [55], reproductive health [56,57], and female health on occupational performance and injury risk [58,59].

Cordell *et al.* [60], screened service records of *n* = 55 (18-41 years) British Army service personnel who had given birth in the last 4 years, and found females were at a significantly increased risk of illness or injury compared to pre-pregnancy. Roach *et al.* [61] carried out a similar study of female US Armed Forces staff, findings showed that those 3-6 months and 12-month post-pregnancy were 7% and 6% respectively, more susceptible to MSK than non-pregnant. Thus, identifying the need for policy and support changes to support service personnel post-partum. Future research should build upon the return-to-work research by Noll *et al.* [62] and explore female-specific return-to-work packages and processes, focusing on post-partum return to work in firefighters.

To date, there is very little physiological data within tactical occupations in the UK in relation to menopause; however, Watkins *et al.* [21], highlighted females’ self-reported concerns around meeting the performance standards pre-, during, and post-menopause. Literature within the general population showed females have found an accelerated decline in aerobic capacity and muscular strength during the menopausal transition [63]. Furthermore, the increases in VO_2_max after training are significantly lower in post-menopausal women compared to peri-menopausal women [64]. Therefore, future research is required to evidence approaches to monitor, maintain and develop fitness during and after the menopausal transition to provide improved support to firefighters to meet the occupational demands of firefighting.

Despite the menstrual cycle [65] and hormonal contraceptives [66] being shown to have a trivial effect on exercise performance, it is widely recognised that there is a large inter-individuality in experiences, often related to the degree of symptomology experienced [67]. In-keeping with other occupations, policies, guidelines and practices could be implemented for individuals with severe dysmenorrhea or menstrual disorders to accommodate menstrual health symptoms where this might affect their physical capability [68] however, further research is needed to understand the extent of this need in female firefighters, in addition to exploring pertinent topics such as whether the known effects of the menstrual cycle on thermoregulation [69] influence exertional heat stress in firefighters.

This is the first study to evaluate the UK-based evidence regarding the assessment, maintenance, and development of physical fitness and occupational performance. As such this has highlighted the limited amount of academic peer-reviewed research that is present with this growing population, including several key areas which contain no available evidence. It is important to note that as this study only used published data, it may not reflect the evidence-base available to the UK fire and rescue service as unpublished, within-service data, may be used to inform guidelines and policies. Whilst this review could have included data from other countries to increase the number of female participants included in the review, this would have reduced the impact of the understanding on performance variables specific to the UK fire and rescue sector. It would be beneficial for future research to use an international data set to compare a larger sample and consider location-specific contexts.

The scoping review highlights the limited understanding of UK female firefighter performance, with only thirty-one separate data sets and nineteen directly measured aerobic capacity values presented. Alongside this, there are only eight sets of simulation performance times published. As such, these limited data sets leave very little understanding of task performance in female firefighters [5,21].

The evidence base shows a limited amount of research around firefighting as an occupation in relation to females, with data potentially underpowered by small sample sizes. Due to the increasing numbers of female firefighters and the desire to increase the diversity of the workforce it is essential that more scientific investigations look at increasing female-specific research increasing the sample size and diversity.

Future studies should look at establishing valid female-specific performance data for cardiorespiratory fitness, muscular strength and endurance and relationships with maximal performance thresholds during firefighting tasks, whilst also exploring the influence of these variables of female performance in firefighting tasks. This information would increase the understanding of female firefighting performance at varying ages and with varying anthropometric measurements [5,21]. This performance data will enable the creation of normative values for female firefighters, developing a clearer understanding of population-specific performance.

Lastly, no published research has been conducted exploring injury rates, or the effects of female-specific considerations (e.g. menstruation, pregnancy and post-partum, and menopause) on fitness assessment or development in UK female firefighters. Therefore, future research is needed to explore injury epidemiology in female firefighters, in addition to identifying the effects of female-specific considerations on the assessment, development and maintenance of physical fitness within the context of the UK fire service. Establishing this information will improve the ability of the UK fire and rescue service to support female firefighters throughout the hormonal and reproductive transitions of their careers.

## Supplementary Material

kqag042_Supplementary_Data
