# Supplementary material for: Scoping review of UK female-firefighters, physical fitness and occupational performance
Source: Occup Med (Lond). 2026 Jun 9;76(4):237–47. doi: 10.1093/occmed/kqag042 (PMC13412390; doi:10.1093/occmed/kqag042)
Supplement: kqag042_Supplementary_Data [file kqag042_supplementary_data.docx]

**Supplementary Data.**

Table 1: Sex specific Firefighter Simulation Assessment performance times and performance variables (Stevenson et al., 2019; Hart et al., 2024).

|  | Female | Male |
| --- | --- | --- |
| Stevenson et al., (2019) | *706 ± 57 | 600 ± 77 |
| Hart et al., (2019) | 632.3 ± 47.6 | 585.6 + 42.1 |
| Mean | *678.4 | 596.7 |
| SD | 62.9 | 70.6 |

*Indicates a significant difference between male and female firefighters established by independent sample T-Tests (p < 0.05

Table 2: Sex specific lower limb strength values absolute (kg) and relative to bodyweight (kg·kg^-1^) for one repetition (1RM) max in the squat movement, for directly measured and estimated (Flex), (Hart et al., 2024)

|  | 1RM (kg) | | | | 1RM kg·kg^-1^ | | | |
| --- | --- | --- | --- | --- | --- | --- | --- | --- |
|  | Male | Female | Male (Flex) | Female (Flex) | Male | Female | Male (Flex) | Female (Flex) |
| Hart et al., 2024 | 139.1 ± 32.5 | 90.6 | 141.2 ± 35.3 | 92.5 | 1.5 ± 0.3 | 1.3 | 1.5 ± 03 | 1.4 |

*One Repetition maximum (1RM), submaximal 1RM estimate (FLEX)
